# Presentation, Treatment, and Outcomes of Non-bacterial Thrombotic Endocarditis in Pancreatic Cancer: A Systematic Review

**DOI:** 10.7759/cureus.104500

**Published:** 2026-03-01

**Authors:** Fahad Hussain, Mahalia Huba, Awais Paracha, Brittany Kwait, Zohair Siddiqui, Humail Patel, Amanda Lee, Himanshu Patel, Sai Palati, Anthony Papale, Robert S Copeland-Halperin, Daniel King

**Affiliations:** 1 Internal Medicine, Donald and Barbara Zucker School of Medicine at Hofstra/Northwell, Manhasset, USA; 2 Internal Medicine, Medical University of South Carolina, Charleston, USA; 3 Internal Medicine, The University of Tennessee Health Science Center, Memphis, USA; 4 Cardiology, Donald and Barbara Zucker School of Medicine at Hofstra/Northwell, Manhasset, USA; 5 Oncology, Donald and Barbara Zucker School of Medicine at Hofstra/Northwell, Manhasset, USA

**Keywords:** hypercoagulable state, malignancy, marantic endocarditis, non-bacterial thrombotic endocarditis, pancreatic cancer, valvular vegetations

## Abstract

Non-bacterial thrombotic endocarditis (NBTE) is characterized by the formation of sterile vegetations on cardiac valves, typically in the setting of malignancy or systemic inflammatory states. These vegetations can cause valvular dysfunction and have a higher tendency to embolize relative to other vegetations, which may result in life-threatening complications. Some of the highest rates of malignancy-associated NBTE have been seen in pancreatic cancer. A review of the literature on pancreatic cancer-associated NBTE was conducted to better understand patient presentation, disease course/progression, treatment, and outcomes. PubMed/MEDLINE was searched using terms related to NBTE and pancreatic cancer. Seventeen studies comprising 19 patients met the inclusion criteria. In 70% of cases, NBTE-related complications prompted the initial diagnosis, whereas pancreatic cancer was the presenting diagnosis in 30%. Valvular involvement most commonly affected the mitral valve (59%), followed by the aortic (32%) and tricuspid (9%) valves. NBTE occurred in the setting of metastatic pancreatic cancer in 68% of patients. Overall mortality was 93% at the time of publication, with death primarily attributable to pancreatic cancer (78%) rather than NBTE itself (22%). Among anticoagulation strategies, mortality was 100% in patients treated with unfractionated heparin and 85.7% in those receiving low-molecular-weight heparin. Given the high mortality and variable clinical picture, there should be a low threshold to initiate NBTE work-up in the presence of pancreatic cancer, regardless of cancer stage.

## Introduction and background

Non-bacterial thrombotic endocarditis (NBTE) is a condition in which sterile tissue deposits, i.e., vegetations, accumulate on cardiac valves, disrupt their function, and pose a risk for embolic events [1,2]. It is a rare condition with rates in autopsy studies ranging from 0.9 to 1.6% in the general population [1,3-5]. NBTE is seen in association with advanced malignancy, systemic lupus erythematosus (SLE), antiphospholipid syndrome, rheumatoid arthritis, sepsis, and burns. The pathogenesis of NBTE is not completely understood, but autopsy studies have shown that these lesions are composed of platelets and fibrin, suggesting they may be formed by endothelial damage in the presence of a hypercoagulable state [6]. The diagnosis is typically made clinically in the presence of valvular vegetations on echocardiogram, after ruling out an infectious etiology. Treatment involves systemic anticoagulation as well as treatment of the provoking disease, causing an underlying hypercoagulable/inflammatory state. Therapeutic doses of subcutaneous low-molecular-weight heparin (LMWH) or intravenous unfractionated heparin (UFH) are the agents of choice for anticoagulation [6,7].

Although NBTE in the general population is rare, rates were found to be higher in patients with pancreatic cancer compared to other malignancy: a study of 1640 patients autopsied over a period of 24 years found patients with adenocarcinoma were at higher risk than patients with other malignant processes (2.70% vs. 0.47%, P < 0.05); especially in cases of pancreatic cancer in comparison with other kinds of adenocarcinoma (10.34% vs. 1.55%, P < 0.05) [3,6]. Furthermore, pancreatic cancer is known to be associated with a higher risk for venous thromboembolism (VTE) relative to other cancers, according to clinically validated tools such as the Khorana Score. Within the Khorana Scoring System, patients are assigned two points for particularly hypercoagulable malignancies (stomach, pancreas) and a single point for lower-risk malignancies (lung, lymphoma, gynecologic, bladder, or testicular) [8,9]. Although further studies are needed to elucidate the mechanism behind pancreatic cancer’s association with NBTE, a higher risk for VTE could portend an overall increased risk of clotting, which may explain why the highest rates of NBTE have been seen amongst patients with pancreatic cancer. However, at this stage, any attempt to explain the association is speculative and hypothesis-driven, as there is no current data to explain the relationship. Given the paucity of literature specifically characterizing NBTE associated with pancreatic cancer, a review was conducted with respect to patient presentation, disease course/progression, treatment, and outcomes to improve our understanding and recognition of this rare but serious condition.

## Review

Methods

Literature Search

This systematic review was registered in the International Prospective Register of Systematic Reviews (PROSPERO) (registration number CRD42024456016). A structured literature search was performed using PubMed/MEDLINE, encompassing publications from database inception through August 26, 2023. Search terms included “non-bacterial thrombotic endocarditis,” “NBTE,” “marantic endocarditis,” "culture negative endocarditis," “sterile endocarditis,” and “Libman-Sacks endocarditis,” each combined in all possible permutations with the terms “pancreatic cancer” and “adenocarcinoma of the pancreas” using the Boolean operator "AND." Searches were restricted to English-language, full-text articles involving human subjects.

Study Selection

**Figure 1:**
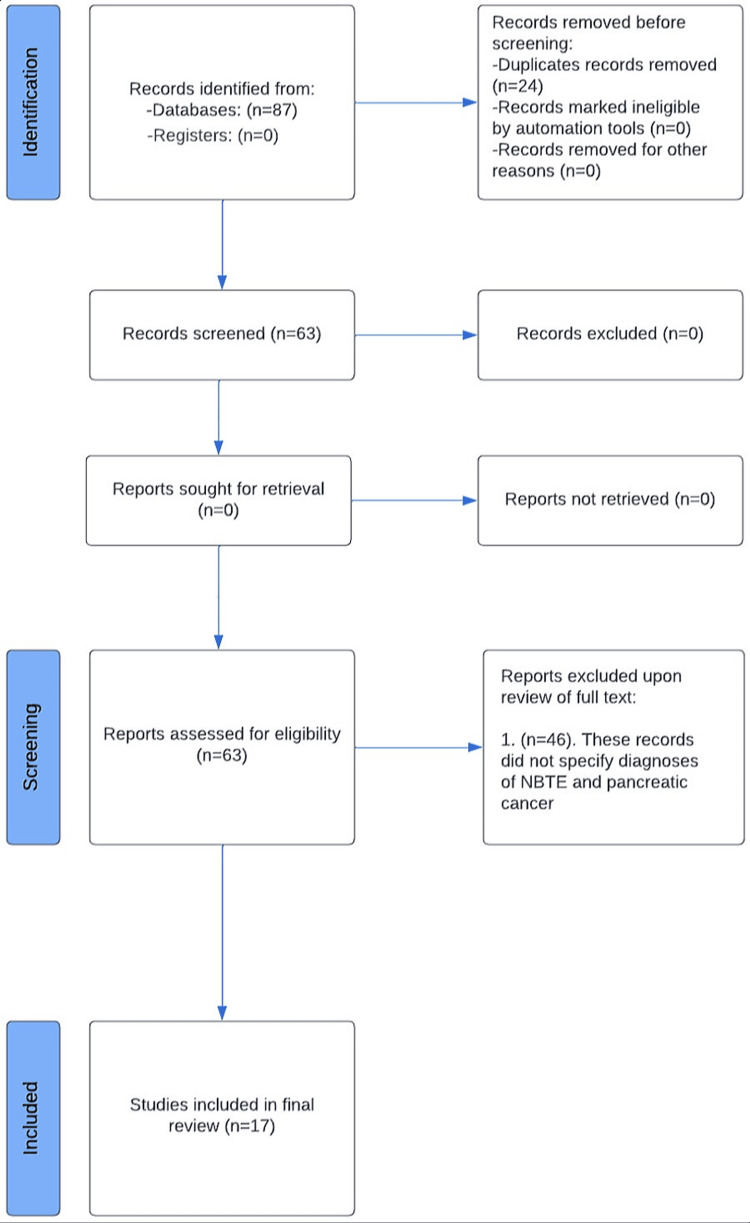
Visual depiction of the search and screening process for all articles included in the review. Eighty-seven records were identified from the PubMed/MEDLINE database, and 24 of these records were removed before screening, as they were duplicate entries. The remaining 63 entries were screened, and 46 were removed as they did not meet the inclusion criteria. Seventeen records encompassing 19 patients were included in the review.

All records identified through the search strategy were independently reviewed by two authors (F.H., A.P.) to assess eligibility (Figure 1). Studies eligible for inclusion were case reports, case series, or other qualitative/quantitative reports describing patients with documented NBTE in association with pancreatic cancer. Articles were excluded if either diagnosis was not clearly established. To reduce the potential for reviewer bias, study selection was conducted independently by both reviewers, with discrepancies resolved through consensus. The systematic review was conducted according to Preferred Reporting Items for Systematic Reviews and Meta-Analyses (PRISMA) guidelines. Assessment of methodological quality and risk of bias was carried out independently by two additional reviewers (Z.S., H.P.) using the Mixed Methods Appraisal Tool (MMAT). No automation software or artificial intelligence tools were employed during study screening, selection, or data analysis.

Data Selection

The primary outcomes evaluated included clinical presentation, diagnostic processes, therapeutic interventions, and mortality among patients with NBTE associated with pancreatic malignancy. Data extracted from each study included patient demographics, medical history, presenting complaints, initial diagnoses, presence and anatomical distribution of organ infarctions, evidence of a hypercoagulable state, metastatic disease status, timing and sequence of NBTE and pancreatic cancer diagnoses, time to diagnosis, physical examination findings, laboratory abnormalities, blood culture results, echocardiographic findings, computed tomography (CT) imaging results, initiation of empiric antibiotic therapy, affected cardiac valves, treatment approaches, overall outcomes, additional diagnostic evaluations, and whether death was attributable to NBTE or pancreatic cancer. All relevant findings corresponding to each data variable were documented. Data extraction was conducted by one author (F.H.) and subsequently reviewed for accuracy by multiple members of the research team. The collected data were analyzed for overarching trends and are presented both narratively and in two summary tables: one outlining patient presentation characteristics and the other detailing diagnostic evaluation features. In cases of absent results for a particular data point, we reported only percentages of the available data, and these instances were explicitly acknowledged in the results section.

Conflict of Interest

The authors report no conflicts of interest related to this study.

Funding

No financial support was received from any funding agencies, institutions, or external sources for this work.

Results

Description of Patients and Neoplasms

This search produced a total of 17 individual case reports from which a total of 19 patients with pancreatic cancer were reported to have experienced NBTE. The patient demographics include eight females (42%) [10-17] and 11 males (58%) [18-26] with an average age of 61 (Table 1).

**Table 1:** Key characteristics regarding patient presentation, diagnosis, treatment, and mortality of all patients included in the review. CVA, cerebrovascular accident; DVT, deep vein thrombosis; LMWH, low-molecular-weight heparin; MI, myocardial infarction; NSTEMI, non-ST-elevation myocardial infarction; PE, pulmonary embolism; TIA, transient ischemic attack; UFH, unfractionated heparin

Paper	Patient	Presenting Diagnosis	Organ Infarcts	Metastasis	Initial Diagnosis (NBTE or Pancreatic Cancer)	Time to Diagnosis (NBTE or Pancreatic Cancer)	Valves Affected	Anticoagulation	Mortality at Study Publication	Cause of Death
Patel et al. [10]	66 y/o female	NSTEMI	Spleen, kidney, brain (CVA)	Liver	NBTE	Unspecified	Mitral, aortic	Unspecified	Expired	NBTE
Wild et al. [11]	67 y/o female	None	None	Liver	Pancreatic cancer	Pancreatic cancer diagnosed two weeks before NBTE	Tricuspid	LMWH	Expired	Pancreatic cancer
Fournier et al. [18]	48 y/o male	Endocarditis	Spleen, kidney	Liver	NBTE	Unspecified	Tricuspid, mitral	LMWH	Expired	Pancreatic cancer
Piovanelli et al. [15]	48 y/o female	CVA	None	None	NBTE	Unspecified	Aortic	UFH	Expired	Pancreatic cancer
Rahman et al. [22]	Middle-aged male	CVA	Spleen, kidney	Liver	NBTE	Unspecified	Mitral	LMWH	Unspecified	Unspecified
Lepour et al. [20]	Male in 60s	CVA	None	Liver	NBTE	Unspecified	Mitral, aortic	LMWH	Expired	Pancreatic cancer
Studdy et al. [12]	58 y/o female	CVA	Lung, kidney, brain (CVA)	None	Unspecified	Unspecified	Mitral	UFH	Unspecified	Unspecified
Starobinska et al. [25]	66 y/o male	CVA	Spleen, kidney, brain (CVA)	None	NBTE	Unspecified	Mitral	Unspecified	Unspecified	Unspecified
Mantovani et al. [16]	65 y/o female	Endocarditis	None	None	NBTE	Unspecified	Mitral, aortic	UFH	Expired	Pancreatic cancer
Randhawa et al. [23]	67 y/o male	CVA	None	Liver	NBTE	Unspecified	Mitral	UFH	Unspecified	Unspecified
Spurgeon et al. [24]	54 y/o male	DVT	Brain (CVA)	None	Pancreatic cancer	Pancreatic cancer diagnosed 10 weeks before NBTE	Mitral	LMWH	Expired	Pancreatic cancer
O'Boyle et al. [21]	56 y/o male	Unspecified	Spleen, brain (CVA)	None	Unspecified	Unspecified	Mitral	None	Expired	Unspecified
Takeshita et al. [14]	65 y/o female	CVA	Brain (CVA)	Liver	Pancreatic cancer	Unspecified	Aortic	UFH	Expired	NBTE
Jameson et al. [19] (first patient reported)	72 y/o male	DVT, PE	Brain (CVA)	Liver	Pancreatic cancer	Pancreatic cancer diagnosed nine months before NBTE	Unspecified	LMWH	Expired	Pancreatic cancer
Jameson et al. [19] (second patient reported)	64 y/o female	CVA	Brain (CVA)	Lung, liver, adrenals	NBTE	Unspecified	Mitral	LMWH	Expired	Unspecified
Jameson et al. [19] (third patient reported)	61 y/o male	DVT, PE	Brain (TIA)	Lung, liver	NBTE	Unspecified	Mitral	LMWH	Alive	N/A
Smeglin et al. [17]	43 y/o female	Pancreatitis	Heart (MI)	Liver	NBTE	Unspecified	Aortic	UFH	Unspecified	Unspecified
Kimyai-Asadi et al. [13]	70 y/o female	Endocarditis	Spleen, heart (MI), brain (CVA)	Liver	NBTE	Unspecified	Aortic	None	Unspecified	Unspecified
Martín Guerra et al. [26]	64 y/o male	DVT, PE	Brain (CVA)	Liver	Pancreatic cancer	Pancreatic cancer diagnosed 72 hours before NBTE	Mitral	LMWH	Unspecified	Unspecified

Presenting Symptoms

The most commonly reported presenting symptoms include neurologic sequelae of brain emboli - weakness, slurred speech, and sensory abnormalities (n = 6) [14,19,20,22,23,26]. Additional symptoms included chest pain (n = 4) [10,12,19,26], altered mental status [15,23,25], weight loss (n = 3) [18,19,21], vision loss (n = 2) [19,23], fatigue (n = 2) [14,17], lower extremity edema (n = 2) [19], and shortness of breath (n = 1) [16]. One case study reported no presenting symptoms [11].

Past Medical History

Of the 19 patient cases, eight reported past medical history. The most commonly reported conditions include a history of tobacco usage (n = 4) [10,22,23,26], hypertension (n = 2) [13,23], pancreatic cancer (n = 2) [11,19], breast cancer (n = 1) [10], type II diabetes mellitus (n = 1) [23], chronic kidney disease (n = 1) [23], deep vein thrombosis (DVT) (n = 1) [23], and heavy alcohol usage (n = 1) [25]. Four of the patient cases were reported to have been previously healthy prior to the diagnosis of pancreatic cancer [15,17-19]. Seven of the patient cases did not specify whether the patients had past medical history [12,14,16,19-21,24].

Diagnostic Presentation

The initial diagnostic presentation included cerebrovascular accident (CVA) (8/18) [12,14,15,19,20,22,23,25], endocarditis (3/18) [13,16,18], DVT (4/18) [19,24,26], pulmonary embolism (PE) (3/18) [19,26], pancreatitis (1/18) [17], and non-ST elevation myocardial infarction (NSTEMI) [10]. One case study did not report a diagnostic presentation, and some studies reported more than one [21].

Diagnostic Work-Up

Diagnostic work-up with echocardiogram was documented in 16 of 19 total cases and was positive for vegetations in 93.8% (15/16) of all specified cases (Table 2) [10,11,13,15-20,22-25]. In the single case in which the echocardiogram was not positive for vegetations, the study was noted to be of poor quality [19]. Transthoracic echocardiography (TTE) alone was used in 12.5% (2/16) [11,18] of specified cases, transesophageal echocardiography (TEE) alone was used in 43.8% (7/16) [11,13,17-19,23] of specified cases, and both TTE and TEE were used in 43.8% (7/16) [10,15,16,20,22,24,25] of specified cases. Additional work-up included negative blood cultures in all specified cases (n = 12) [10,13,15-20,22-25] and a CT scan consistent with pancreatic cancer in all specified cases (n = 15) [10,13-20,22-26].

**Table 2:** Relevant diagnostic work-up, including imaging findings and blood culture results for all patients included in the review. AS, aortic stenosis; AR, aortic regurgitation; AV, aortic valve; CT A/P, computed tomography of the abdomen and pelvis; LVEF, left ventricular ejection fraction; MR, mitral regurgitation; MV, mitral valve; RA, right atrium; TEE, transesophageal echocardiogram; TTE, transthoracic echocardiogram; TV, tricuspid valve

Paper	Patient	Blood Culture	Echocardiogram	CT A/P Findings
Patel et al. [10]	66 y/o female	No growth	TTE - LVEF of 33%, severe MR, multiple mobile echodensities on MV, severe AS; TEE - multiple mobile masses on MV and AV	Pancreatic head mass measuring 2.3 cm, multiple splenic and renal infarcts, low-density masses in the liver consistent with metastases
Wild et al. [11]	67 y/o female	Unspecified	TTE - large masses adherent to TV leaflets	Unspecified
Fournier et al. [18]	48 y/o male	No growth	TEE - mobile, echogenic foci on TV and MV	Mass in the neck of the pancreas, multiple liver lesions, hypodensities in the spleen and kidneys consistent with infarcts
Piovanelli et al. [15]	48 y/o male	No growth	TTE/TEE - multiple vegetations on AV, moderate AR	Mass in the tail of the pancreas
Rahman et al. [22]	Middle-aged male	No growth	TTE - low-normal LVEF, mild MR; TEE - MV vegetation	Bilateral pulmonary embolism, evidence of kidney and spleen infarction, a mass in the tail of the pancreas, and multiple liver metastases
Lepour et al. [20]	Male in 60s	No growth	TTE/TEE - vegetations on MV, AV with severe MR and moderate AR	Pancreatic tumor with multiple liver metastases
Studdy et al. [12]	58 y/o female	Unspecified	Unspecified	Unspecified
Starobinska et al. [25]	66 y/o male	No growth	TTE - negative; TEE - vegetation on the posterior leaflet of the MV	Dilation of the main pancreatic duct and a mass in the pancreatic head
Mantovani et al. [16]	65 y/o female	No growth	TTE - severe AR, MR; TEE - multiple mobile vegetations on AV and MV	Multiple lesions of the pancreas, suspicious for malignancy
Randhawa et al. [23]	67 y/o male	No growth	TEE - multiple echogenic densities of MV	Bilateral pulmonary emboli, a heterogeneous mass at the distal body/tail of the pancreas, with metastatic disease found throughout the liver
Spurgeon et al. [24]	54 y/o male	No growth	TTE - negative; TEE - small echogenic mass lesions on tips of MV leaflets	Hypodense mass in the body of the pancreas, extensive liver metastases, probable peritoneal deposits
O'Boyle et al. [21]	56 y/o male	Unspecified	Unspecified	Unspecified
Takeshita et al. [14]	65 y/o female	Unspecified	Unspecified	Pancreatic tumor with multiple metastases
Jameson et al. [19] (first patient reported)	72 y/o male	Unspecified	TEE - poor quality of study, but negative for vegetations	Unspecified
Jameson et al. [19] (second patient reported)	64 y/o male	Unspecified	TEE - vegetation on MV	Mass in the head of the pancreas; multiple liver, lung, and adrenal lesions
Jameson et al. [19] (third patient reported)	61 y/o male	Unspecified	TEE - string thrombus in transit across the intra-atrial septum	Mass in the tail of the pancreas with multiple metastatic lesions in the liver and lungs
Smeglin et al. [17]	43 y/o female	No growth	TEE - multiple vegetations of AV with moderate AR	Diffuse enlargement of the body of the pancreas with a low-density mass
Kimyai-Asadi et al. [13]	70 y/o female	No growth	TEE - left ventricular hypertrophy, several vegetations of AV with moderate AR, large RA thrombus	Irregular mass in the tail of the pancreas with several lesions throughout the liver consistent with infarctions or metastases; several splenic infarctions
Martín Guerra et al. [26]	64 y/o male	No growth	TTE - severe mitral insufficiency, masses on MV	Mass in the body of the pancreas and multiple liver metastases

Total organ infarcts over the duration of stay included CVA (n = 14) [10,12-15,19-26], splenic infarcts (n = 6) [10,13,18,21,22,25], kidney infarcts (n = 5) [10,12,18,22,25], and myocardial infarction (n = 2) [13,17]. Five patient cases reported no organ infarcts [11,15,16,20,23], and seven cases reported multiple [10,12,13,18,21,22,25]. Other indications of a hypercoagulable state included DVT (n = 9) [11,12,16,19,22-24,26], PE (n = 7) [12,16,19,22,23,26], and disseminated intravascular coagulation (DIC) (n = 1) [17]. In terms of testing done for hypercoagulable state, D-dimer was elevated in 100% (6/6) of cases in which it was reported [11,14,16,22,24,26], and antiphospholipid antibodies were negative in 100% (6/6) cases in which they were reported [10,13,15,17,20,22].

Valvular Localization

Of the 19 patients in this study, 18 were found to have evidence of valvular vegetations by echocardiogram or autopsy. Among these 18 patients, there were 22 valves involved, most commonly the mitral valve at 59.1% (13/22) [10,12,16,18,20-26], followed by the aortic valve at 31.8% (7/22) [10,13-17,20], and then the tricuspid valve at 9.1% (2/22) [11,18]. Four patients had multi-valvular disease, with the aortic-mitral combination (n = 3) [10,16,20] being the most common, followed by tricuspid-mitral (n = 1) [18]. One patient was not found to have echocardiographic evidence of vegetations, and autopsy findings were not specified [19].

Time to Diagnosis of NBTE/Pancreatic Cancer

Of the 19 cases in this study, 17 specified a diagnostic timeline. In these cases, NBTE (n = 12) [10,13,15-20,22,23,25,26] was predominantly diagnosed before pancreatic cancer (n = 5) [11,14,19,24,26].

Metastasis

Metastasis was reported in 68.4% (13/19) of cases [10,11,13,14,17-20,22,23,26]. Of these 13 cases with reported metastases, three did not specify patient mortality at study publication [17,23,25]. In the 10 cases of metastatic malignancy in which mortality was reported, 90% (9/10) of patients had expired by the time of study publication [10,11,13,14,18-20,22]. In cases where metastasis was reported, the most common site of metastasis was the liver (n = 13) [10,11,13,14,17-20,22,23,26]. The other reported sites included the lung (n = 2) [19] and adrenal glands (n = 1) [19]. Of the six patients without metastases, the overall mortality rate, at publication of the study, was 100% (5/5) [12,15,16,21,24], with one patient case not reporting outcome [25]. Patients with metastases to the liver had a mortality rate of 90% (9/10 expired), patients with metastases to the lungs had a 50% (1/2 expired) [19] mortality rate, and patients with metastases to the adrenals had a 100% (1/1 expired) [19] mortality rate. 

NBTE Treatment

Treatment type (including no treatment) was specified in 17 of 19 patient cases. Treatment primarily focused on anticoagulation (n = 17) [10-12,14-20,22-26], with LMWH (n = 9) [11,18-20,22,24,26] being the most common agent, followed by UFH (n = 6) [12,14,15,16,17,23]. Two patient cases reported that no treatment was given [13,21]. In one of those cases, the patient expired before treatment could be initiated, and the patient in the other case was put on comfort measures before treatment could be initiated. 

Mortality was documented in three of six patients treated primarily with UFH and seven of nine patients treated predominantly with LMWH. Of the patients primarily treated with UFH, mortality was 100% (3/3 patients expired at study publication) [11,14,21], with three patient cases not specifying outcome [17,23]. In patients treated primarily with LMWH, mortality was 85.7% (6/7 patients expired, 1/7 alive at study publication) [11,18-20,24], with two patient cases not specifying outcome [22,26].

Summarization of Causes of Death

Of the 19 patient cases, 14 patients were reported dead [10-16,18-22,24], and one patient was reported alive [19], while four did not specify the patient outcome [17,23,25,26]. Of the 14 patient cases that reported a death outcome, only nine reported a cause of death, which were sequelae of pancreatic cancer in 77.8% (7/9) [11,15,16,18-20,24] and embolic stroke secondary to NBTE in 22.2% (2/9) [10,14].

Discussion

NBTE is a rare condition frequently associated with advanced malignancy, with some of the highest rates seen in pancreatic cancer. Definitive diagnosis of NBTE is made pathologically, but due to the invasive nature of valvular biopsy, this is not typically done. Instead, the diagnosis of NBTE is frequently made due to high clinical suspicion in the setting of echocardiographic findings, underlying malignancy, and an absence of microbiologic or other evidence of systemic infection [7]. Making the diagnosis of pancreatic cancer-associated NBTE can be challenging, as history and physical exam findings are often nonspecific and variable. Our data suggests that patients typically present with symptoms that are the direct result of thromboembolic phenomena (motor weakness, slurred speech, altered mental status, pleuritic chest pain, shortness of breath, lower extremity edema) or malignancy (weight loss, fatigue). These are broad categories that can result in a variety of different clinical presentations. Furthermore, although past medical history such as tobacco use, hypertension, diabetes mellitus, malignancy, and alcoholism are reported in some patient cases, they are non-specific, and, in 33% of patient cases, there was no past medical history.

Initial diagnosis at presentation in patients with pancreatic cancer-associated NBTE was predominantly CVA (44% of patient cases), followed by DVT/PE (22% of patient cases) and valvular vegetation (17%). This suggests that thromboembolic symptoms are often the first to materialize in pancreatic cancer-associated NBTE. Unexplained thromboembolism should guide the clinician towards a hypercoagulable work-up, which can lead to the diagnosis of malignancy in the appropriate patient population. Additionally, given that metastatic pancreatic cancer was found in 68% of patient cases, it is interesting to note that it was not the initial diagnosis in any of our patient cases. Rather, pancreatic cancer was diagnosed later in the hospital course, and after NBTE was diagnosed in most cases. However, it was predominantly listed as the cause of death, lending credence to the idea of pancreatic cancer as a particularly insidious malignancy.

Given that most patients presented with neurologic symptoms and were diagnosed with CVA, it is easy to understand why NBTE was predominantly diagnosed before pancreatic cancer. In patients diagnosed with acute ischemic stroke, echocardiography is a recommended part of the work-up to rule out a cardio-embolic source [27]. Additionally, pancreatic cancer often causes vague, subclinical symptoms and is typically diagnosed late in the disease course. Valvular lesions in the studied cases of pancreatic cancer-associated NBTE most frequently involved the mitral valve (59%), followed by the aortic valve (32%) and then the tricuspid valve (9%). Although exact figures vary by study, this data is similar to data on NBTE in cancer patients, which shows valvular lesions predominantly on the mitral valve, followed by the aortic, and then the right-sided valves [28,29]. This distribution is also similar to that seen in infective endocarditis, most commonly showing involvement of left-sided heart valves, particularly the mitral valve [30,31]. This distribution is theorized to be due to left-sided valves being exposed to higher pressures and therefore being at a higher risk for underlying structural damage, which then predisposes them to vegetation formation [30].

The vegetations of NBTE are often small and may not be visible on TTE [32]. In these instances, in the presence of adequate clinical suspicion for the condition, TEE can be a more sensitive test. In our review, TTE was negative for vegetations, which were later picked up on TEE in two patient cases. In one case, both TTE and TEE were negative, but the patient was anticoagulated due to otherwise high clinical suspicion for a hypercoagulable state. In cases with negative or equivocal echocardiographic results and suspected embolic CVA, the stroke pattern can be used to increase or decrease clinical suspicion for NBTE. NBTE typically exhibits a pattern of numerous lesions in multiple cerebral territories, varying greatly in size [33]. Additionally, compared to the vegetations seen in infective endocarditis, the vegetations of NBTE are smaller and more easily displaced, leading to dislodgment with devastating embolization [34].

A majority (68%) of the patient cases reported metastatic disease associated with pancreatic cancer. Our data support NBTE’s association with advanced malignancy, a form of hypercoagulable state [35,36]. However, as 32% of pancreatic cancer-associated NBTE cases occurred in the absence of metastatic malignancy, it is important to note that even patients with localized pancreatic cancer manifest a hypercoagulable state. Given the dire consequences of NBTE, further studies are needed to elucidate risk factors associated with the condition to create a risk-assessment tool or otherwise screen patients with high-risk forms of malignancy for NBTE.

There was no significant difference between the mortality rate at study publication in patients with NBTE and metastatic pancreatic cancer and those with NBTE and pancreatic cancer without metastases. As mortality rates were nearly 100% in both scenarios, this is likely a reflection of poor overall prognosis in pancreatic cancer complicated by NBTE. All-cause mortality in patients with pancreatic cancer-associated NBTE at study publication was high, at 93%. Interestingly, although patients typically presented with embolic complications from NBTE, the listed causes of death were predominantly due to sequelae of pancreatic cancer (78%). This discrepancy might be a result of many of our patients presenting with advanced pancreatic malignancy, which, itself, has a poor prognosis and outcomes [37,38]. It might also be indicative of the effectiveness of systemic anticoagulation in prophylaxis of NBTE-associated embolic events.

Management of NBTE typically consists of treatment of the underlying malignancy and systemic anticoagulation with the therapeutic dose of LMWH or UFH [6,7]. Valve surgery can be considered on a case-by-case basis in patients in whom the benefits outweigh the risks. This includes patients with appropriate functional status, a high degree of valvular dysfunction, recurrent embolic events on anticoagulation, and patients without advanced malignancy [28,39]. Patients in our study population are often excluded from consideration for valvular surgery due to poor prognosis from their primary disease. In our review of the literature, treatment was only with anticoagulation, and valvular surgery was never attempted. LMWH was the most used anticoagulant. Mortality was slightly lower in the group treated with LMWH (85.7%) as compared to the group treated with UFH(100%). However, given the small sample size and the prevalence of advanced pancreatic cancer in this group, no definitive conclusions about the efficacy of UFH compared to LMWH can be drawn.

An interesting avenue for future research lies in examining the role of direct oral anticoagulants (DOACs) in the treatment and prevention of NBTE. DOACs have been studied as thromboprophylaxis for patients with active cancer and at an increased risk of developing VTE [40]. Findings indicated that apixaban therapy resulted in a significantly lower rate of VTE among ambulatory patients with cancer at intermediate-to-high risk (per Khorana Scoring) of developing VTE compared with placebo [41]. Without further studies, it is impossible to predict whether DOAC thromboprophylaxis would also result in lower rates of NBTE in known cancer, but given the high mortality associated with the condition, further investigation is warranted. Given that the majority of patients in our review presented before pancreatic cancer had been diagnosed, another area of exploration could be treatment outcomes in NBTE with DOACs as opposed to UFH and LMWH.

This study is limited primarily by the lack of literature examining NBTE in patients with associated pancreatic cancer. Our review of the literature yielded only case reports, with 19 patient cases. Since this review uses secondary data, the availability of some data and the quality of the study design could not be completely controlled. Taken together, these limitations impact our study’s ability to confidently analyze trends in NBTE associated with pancreatic cancer and reflect the limited knowledge regarding the entity. Furthermore, our study design excludes databases aside from PubMed/MEDLINE, non-English language articles, and studies that are not full manuscripts, which might limit the total body of literature examined in our study. Future studies should focus on a case-control design aiming to identify risk factors for the development of NBTE.

## Conclusions

NBTE reflects a hypercoagulable state and often arises in the setting of an advanced malignancy. In patients with pancreatic cancer presenting with embolic symptoms, the threshold for NBTE workup should remain low due to the high mortality of this disease. Higher-powered studies are needed in the future to develop a comprehensive patient risk profile to prophylactically screen and identify high-risk patients. With the development of DOACs, future studies are needed to examine if there are additional or alternative treatments for NBTE in pancreatic cancer patients to improve mortality in this patient population. 
